# Synthesis of Porous Materials on Hybrid Wormlike Micelles of Zwitterionic and Anionic Surfactants for Efficient Oilfield Wastewater Treatment

**DOI:** 10.3390/gels11090714

**Published:** 2025-09-05

**Authors:** Fei Liu, Zhenzhen Li, Chenye Yang, Ya Wu, Ying Tang

**Affiliations:** 1 College of Petroleum Engineering, Shandong Institute of Petroleum and Chemical Technology, Dongying 257061, China; 2 Efficient Exploration and Development of Oil and Gas Reservoirs and the Integration of Geology and Engineering Shandong Provincial Engineering Research Center, Dongying 257061, China; 3 Shaanxi Province Key Laboratory of Environmental Pollution Control and Reservoir Protection Technology of Oilfields, Xi’an Shiyou University, Xi’an 710065, China; 23212071065@stumail.xsyu.edu.cn (Z.L.); 23211071020@stumail.xsyu.edu.cn (C.Y.); wuya@xsyu.edu.cn (Y.W.)

**Keywords:** sulfonated lignite, hierarchical hydrotalcite, anionic/zwitterionic wormlike micelles

## Abstract

Addressing the challenge of sulfonated lignite (SL) removal from oilfield wastewater, this study introduces a novel hierarchical MgFe-layered double hydroxide (LDH) adsorbent. The material was fabricated via in situ co-precipitation, utilizing a template formed by the NaCl-induced co-assembly of oleylaminopropyl betaine (OAPB) and sodium dodecyl sulfate (SLS) into zwitterionic, anionic, shear-responsive viscoelastic gels. This gel-templating approach yielded an LDH structure featuring a hierarchical pore network spanning 1–80 nm and a notably high specific surface area of 199.82 m^2^/g, as characterized by SEM and BET. The resulting MgFe-LDH demonstrated exceptional efficacy, achieving a SL removal efficiency exceeding 96% and a maximum adsorption capacity of 90.68 mg/g at neutral pH. Adsorption kinetics were best described by a pseudo-second-order model (R^2^ > 0.99), with intra-particle diffusion identified as the rate-determining step. Equilibrium adsorption data conformed to the Langmuir isotherm, signifying monolayer uptake. Thermodynamic analysis confirmed the process was spontaneous (ΔG < 0) and exothermic (ΔH = −20.09 kJ/mol), driven primarily by electrostatic interactions and ion exchange. The adsorbent exhibited robust recyclability, maintaining over 79% of its initial capacity after three adsorption–desorption cycles. This gel-directed synthesis presents a sustainable pathway for developing high-performance adsorbents targeting complex contaminants in oilfield effluents.

## 1. Introduction

As oil extraction activities increase in volume, a concomitant rise in the production of industrial wastewater is inevitable during the exploration and development phases of crude oil. This wastewater contains a variety of environmentally persistent organic pollutants characterized by large molecular weights, posing significant degradation challenges. Sulfonated lignite, employed as a sulfonated lignite mud treatment agent, serves to mitigate filtration loss and enhance the temperature resistance of drilling fluids [1]. Nevertheless, the elimination of sulfonated lignite from wastewater streams proves arduous due to the presence of negatively charged sulfonic groups. These groups augment the pollutant’s affinity for binding with other substances in water, complicating their separation during wastewater treatment processes [2]. Adsorption involves the reversible replacement of ions between a solid adsorbent (e.g., zeolites, resins) and a liquid/gas phase has been identified as a particularly promising approach for the economical and straightforward removal of organic pollutants, given the range of treatment methodologies available [3]. Various adsorbents, including activated carbon, zeolites, natural clay materials, and metal oxides, have undergone extensive investigation for their pivotal roles in adsorbing pollutants from aqueous solutions [4,5,6,7]. However, the simplistic pore architecture of many conventional adsorbents renders them inadequate for capturing macromolecular organic pollutants, such as sulfonated lignite. Hydrotalcites, a class of layered double hydroxides, are structured with positively charged metal hydroxide layers and interlayer exchangeable anions arranged in a specific stoichiometric ratio, forming a highly ordered layered configuration [8]. Owing to their expansive specific surface area and remarkable thermal stability, hydrotalcites find broad applications in catalysis, adsorption, and ion exchange domains [9]. Ogata studied the adsorption capacity of magnesium-iron hydrotalcite with different molar ratios (Mg/Fe = 3.0 and 5.0) for phosphates in water, explaining that the adsorption mechanism is mainly related to ion exchange and electrostatic attraction [10]. It was found that Mg^2+^ in the hydrotalcite provides structural stability through strong ionic bonding in the brucite-like layer, preventing lattice collapse during anion exchange and Fe^3+^ introduces Lewis acidity and a higher positive charge density, enhancing electrostatic attraction toward anionic pollution molecules. Nonetheless, the constrained interlayer spacing of conventional hydrotalcites impedes the ingress of macromolecules or polymers, thereby limiting their efficacy in the removal of macromolecular organic pollutants.

Consequently, the development of multistage pore hydrotalcites with enlarged specific surface areas, intricate pore networks, and superior adjustability becomes imperative for the efficient treatment of macromolecular organic pollutants [11]. In this study, multigrade hydrotalcites were synthesized utilizing wormlike micelles (WLMs) as soft templates. As the concentration of additives (salts or organic acids) escalates, the viscosity of the micellar solution peaks, prompting the micelles to entangle and interconnect, forming WLMs with a three-dimensional meshwork [12,13]. These WLMs, characterized by their transient network structure, exhibit viscoelastic properties akin to those of polymeric fluids, albeit with a constant dynamic process of breakage and reformation, earning them the moniker “living polymers” [14]. Currently, WLMs are primarily constructed from cationic surfactants due to their strong thickening capabilities, whereas the use of anionic surfactants has been limited by their relatively poor thickening performance. However, WLMs based on mixtures of zwitterionic and anionic surfactants have been extensively investigated [15,16]. Notably, the viscoelastic properties of OAPB and SDS have been well documented [17] and zwitterionic/anionic WLMs have demonstrated significant potential as templates for directed nanomaterial synthesis [18]. Hence, the formation of heterogeneous WLMs through the combination of zwitterionic and anionic surfactants presents a viable and common approach [19]. In our investigation, sodium chloride (NaCl) was employed as an additive to facilitate the self-assembly of zwitterionic surfactant oleyl aminopropyl betaine (OAPB) and anionic surfactant sodium dodecyl sulfate (SLS) into entangled hybrid WLM aggregates. These aggregates served as soft templates for the in situ growth of MgFe hydrotalcite via co-precipitation, resulting in well-dispersed hierarchical structures with an increased number of active sites. This structural enhancement aimed to augment the adsorption capacity for macromolecular organic pollutants. Detailed examinations were undertaken to elucidate the adsorption kinetics and thermodynamics, along with an exploration of the underlying adsorption mechanism.

## 2. Result and Discussion

### 2.1. Preparation of Wormlike Micelles

**Figure 1:**
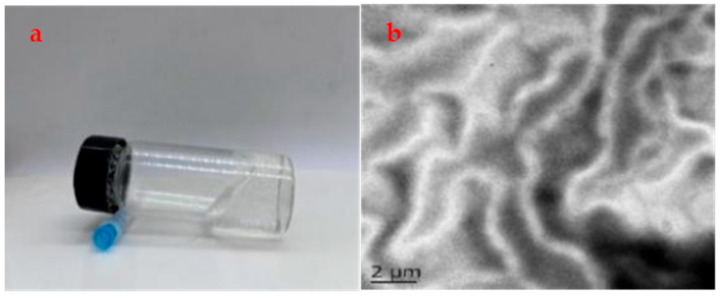
(**a**) Appearance and (**b**) low-pressure transmission electron microscopy (LVTEM) of viscoelastic fluid.

Dissolve appropriate amount of OAPB and SLS in 0.1 mol/L NaCl which was introduced to reduce intermolecular electrostatic repulsion through charge shielding under stirring to obtain gel samples [20]. As shown in Figure 1a, the small molecule aqueous micellar gel prepared at room temperature has high viscosity due to the electrostatic attraction between the anionic SLS head group and the OAPB quaternary ammonium salt group, and it can also maintain a non-flowing posture when dumped. The viscoelastic fluid was freeze-dried at low temperature and characterized by TEM. It can be seen that the modified small molecule viscoelastic fluid has a typical three-dimensional network structure (Figure 1b).

### 2.2. Formation of Wormlike Micelles

**Figure 2:**
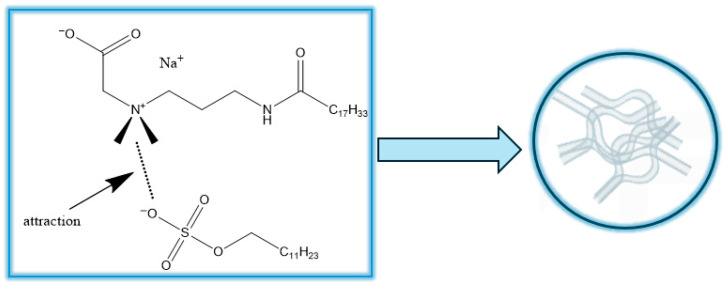
Formation mechanism of wormlike micelles.

The formation of viscoelastic fluid formed by viscoelastic wormlike micelles may be attributed to the deprotonation of carboxyl groups in OAPB (Figure 2). The hydroxyl ion (OH^−^) promotes the deprotonation of the carboxyl group of OAPB, enhances the electrostatic attraction between the surfactant head groups, and thus accelerates the formation of gel network by strengthening micellization [21]. However, the morphology and size of micelles are still limited by intermolecular electrostatic repulsion. After adding sodium chloride, Na^+^ and Cl^−^ ions interact with charged sites on OAPB and SLS molecules, inducing charge shielding to reduce electrostatic repulsion and increase the strength of micelle entanglement, thereby macroscopically improving viscosity.

### 2.3. Rheological Behavior of Wormlike Micelles

**Figure 3:**
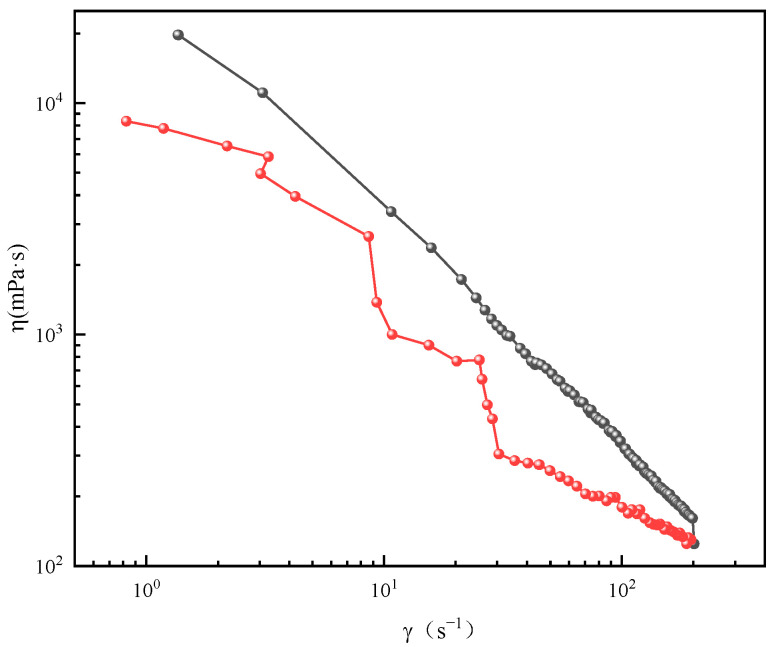
Plot of shear rate versus viscosity.

By analyzing the correlation between shear rate and apparent viscosity (as depicted in Figure 3), it can be seen that as the shear rate of the solution increases, its apparent viscosity exhibits a corresponding decrease. This shear-thinning behavior is primarily attributed to the alignment of the long wormlike micelles along the direction of flow under high shear conditions, which reduces their resistance to flow [17]. Conversely, when the shear rate diminishes, the apparent viscosity of the micellar solution gradually recovers and rises. This behavior underscores the presence of wormlike micelles within the solution, which are characterized by their intertwined and entangled mesh-like structures [22].

**Figure 4:**
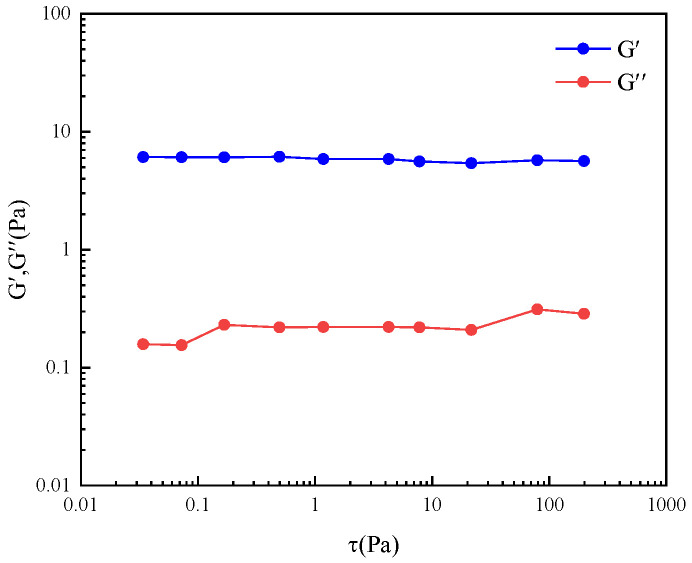
Linear Viscoelastic zone of SLS/OAPB wormlike micelles.

As shown in Figure 4, the material exhibits stable G′ values around 10 under various stresses (τ, Pa), indicating minimal variation in its elastic properties within the tested stress range. G″ exhibits slight fluctuations at low stresses, with values ranging from 0.1 to 0.5. As stress increases, G″ shows a slight upward trend, though overall variation remains minimal. This indicates that the material’s viscous properties also remain relatively stable within the tested stress range. These findings suggest the material exhibits good linear viscoelastic behavior within the tested stress range [23].

**Figure 5:**
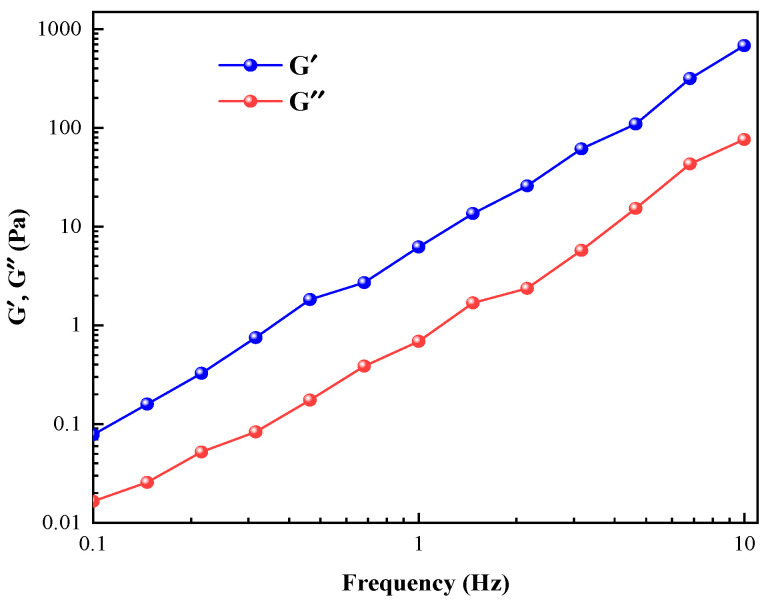
SLS/OAPB worm-like micelle viscoelasticity.

Figure 5 illustrates the variation in dynamic moduli (G′ and G″) in the material across different frequencies. The horizontal axis represents frequency (Hz), while the vertical axis shows the values of dynamic moduli (G′ and G″) in Pa. The blue and red lines in the figure depict the trends of G′ and G″, respectively. As shown, both G′ and G″ values gradually increase with rising frequency. This indicates that both the elastic modulus and loss modulus of the material increase with frequency. In the low-frequency range, the values of G′ and G″ are relatively small, while in the high-frequency range, they increase significantly. This may occur because at low frequencies, the material’s internal structure has sufficient time to respond to changes in external force, whereas at high frequencies, the internal structure cannot respond in time, leading to an increase in modulus. Furthermore, the figure shows that G′ consistently exceeds G″, indicating the elastic modulus remains greater than the loss modulus. This likely stems from the material’s internal structure primarily exhibiting elastic deformation under external forces, with loss deformation being comparatively minor [24].

### 2.4. Characterization of MgFe-LDH Samples

**Figure 6:**
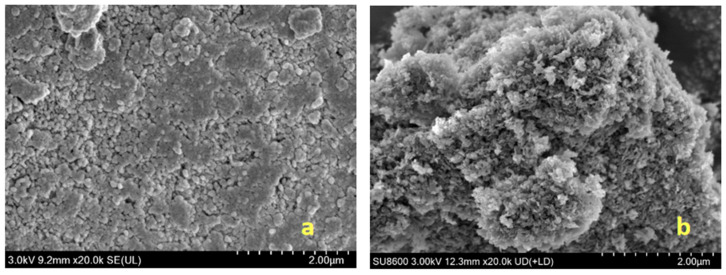
SEM image of hydrotalcite ((**a**) hydrotalcite; (**b**) hierarchical hydrotalcite).

As illustrated in Figure 6a, the MgFe-LDH synthesized via the co-precipitation technique displays a well-defined hexagonal morphology, accompanied by a relatively narrow distribution in lateral dimensions. In contrast, Figure 6b reveals that the W-MgFe-LDH, which was fabricated utilizing wormlike micelles as soft templates, demonstrates an interwoven morphological feature. This indicates the formation of a hierarchical architecture characterized by a regular, layered structure [25].

**Figure 7:**
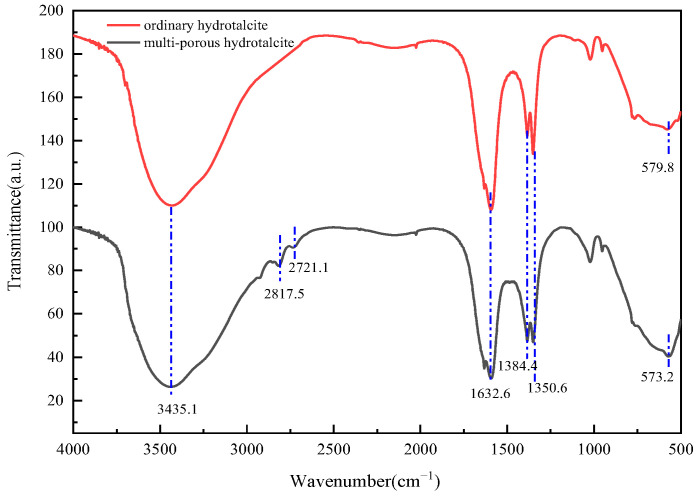
FTIR spectra of MgFe-LDH.

Examination of Figure 7 reveals that the infrared spectral profiles of hierarchical hydrotalcite and conventional hydrotalcite exhibit striking similarity. Nevertheless, two distinct absorption bands at 2721.1 and 2817.5 cm^−1^ are discernible in the hierarchical hydrotalcite W-MgFe-LDH, which can be ascribed to the bending vibrations of CH_2_ groups. These vibrations arise due to the incorporation of surfactants within the wormlike micellar templates [26]. The broad and intense absorption peak centered at 3435.1 cm^−1^ is indicative of the stretching vibrations of O-H bonds and water molecules situated within the hydroxide layers. Additionally, the absorption band located near 1384.4 cm^−1^ corresponds to the asymmetric stretching vibrations of C-O bonds, suggesting the presence of carbonate anions within the MgFe-LDH structure. Furthermore, the absorption band spanning the range of 900–400 cm^−1^ is attributed to the stretching vibrations of metal-oxygen bonds, metal-hydroxy bonds, and the deformation vibrations of metal-oxygen bonds within the LDH layers [27].

**Figure 8:**
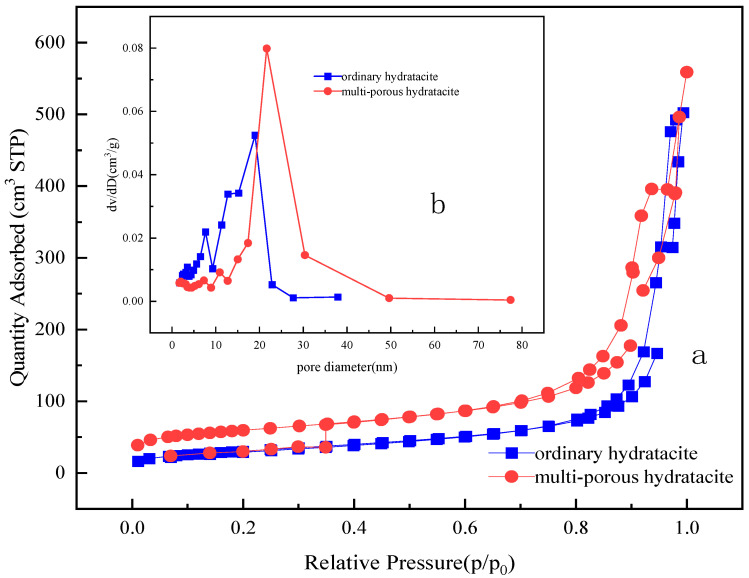
The N_2_ adsorption–desorption isotherms (**a**) and pore size distribution curve of MgFe-LDH (**b**).

The N_2_ adsorption–desorption isotherms along with the pore size distributions for both MgFe-LDH and W-MgFe-LDH were systematically evaluated. As depicted in Figure 8a, the N_2_ adsorption–desorption isotherms for both samples conform to Type IV patterns, which is indicative of the presence of mesoporous structures. Notably, a distinct hysteresis loop is observed in the isotherms of W-MgFe-LDH, suggesting an enhanced adsorption capacity at elevated relative pressures (p/p_0_ > 0.5), a characteristic attributed to the existence of macropores [28]. In comparison to traditional hydrotalcite, hierarchical hydrotalcite exhibits a broader pore size distribution, spanning from 1 nm to 80 nm, as illustrated in Figure 8b. The BET analysis results for MgFe-LDH, presented in Table 1, further substantiate the meso-macroporous nature of W-MgFe-LDH. Moreover, the incorporation of colloidal templates resulted in a significant augmentation of the BET surface area (increasing from 110.23 to 199.82 m^2^/g) and pore volume (expanding from 0.63 to 0.78 cm^3^/g), thereby offering substantial potential for superior adsorption performance. It is noteworthy that the reduction in pore size observed for W-MgFe-LDH can be rationalized by the proliferation of pore numbers per unit volume.

**Figure 9:**
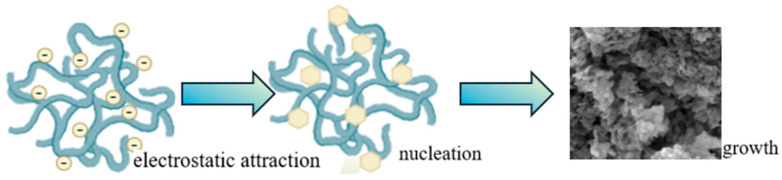
The formation process of multi-stage channel hydrotalcite.

It is well-established that the synthesis of hydrotalcite involves a heterogeneous crystal nucleation process [29,30]. Under alkaline circumstances, as the metal solution is gradually added drop-by-drop, amorphous Fe hydroxide is initially formed. Subsequently, Mg hydroxide starts to deposit (as illustrated in Figure 9). During this process, free Fe^3+^ ions diffuse into the Mg(OH)_2_ layer structure and substitute Mg^2+^ ions, resulting in the crystal nuclei carrying a positive charge. To maintain charge neutrality, anions such as OH^−^ and CO_3_^2−^ are incorporated into the hydrotalcite layers.

In the context of using a wormlike micelle solution (which can be considered a type of gel-like system with unique structural and functional properties), the behavior of anions is distinct. These anions tend to ionize and adsorb onto the surface of the hydrotalcite crystal nuclei. The presence of the wormlike micelle-based gel environment plays a crucial role here. The gel’s structure provides a specific micro-environment that facilitates the adsorption of anions on the crystal nuclei. This adsorption phenomenon significantly reduces the likelihood of crystal nucleus agglomeration. By preventing agglomeration, the wormlike micelle-based gel helps in achieving a more uniform and well-dispersed hydrotalcite product with potentially improved properties for various applications.

### 2.5. Preparation Parameters of Hydrotalcite

**Figure 10:**
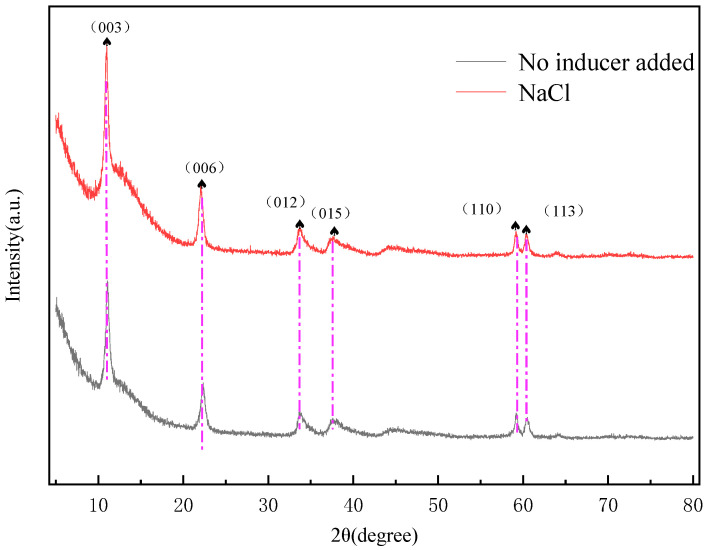
Effect of the type of inducer of micelles on MgFe-LDH.

As illustrated in Figure 10, the hydrotalcite synthesized using micelles as soft templates exhibits a maximum full-width at half-maximum (FWHM) value of 0.752° for its diffraction peak. when NaCl is employed as an inducer, the FWHM of the diffraction peak becomes even more constricted, measuring 0.409°. This narrowing of the FWHM implies an augmentation in the intensity of the diffraction peak, an enhancement in crystallinity, and an optimization of the regularity of the crystal structure [31]. Considering the distinctive network-like structural morphology of wormlike micelles, when hydrotalcite is formed under in situ generation conditions with wormlike micelles serving as templates, it can demonstrate superior dispersibility and a higher degree of crystallinity. The unique structural features of wormlike micelles provide a favorable micro—environment that influences the growth and arrangement of hydrotalcite crystals, leading to these improved properties.

**Figure 11:**
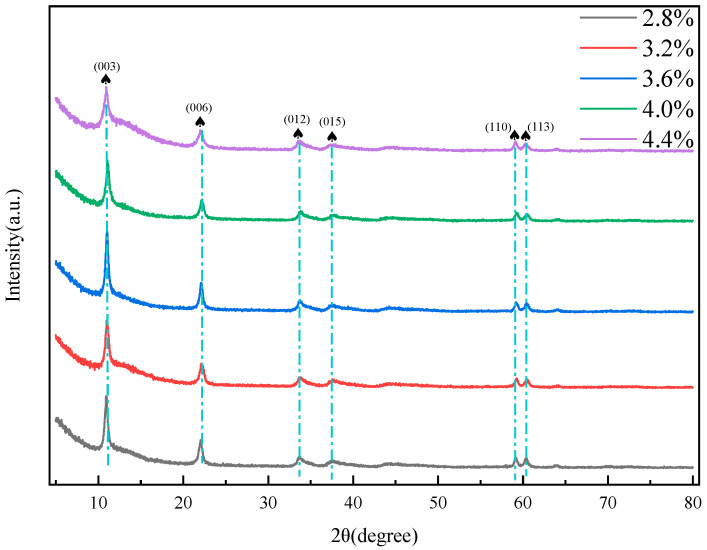
The XRD patterns of the prepared MgFe-LDH derived from surfactant solutions of different concentrations.

As depicted in Figure 11, the MgFe-LDHs synthesized at varying total surfactants concentrations exhibit the characteristic crystal planes typical of hydrotalcite. When the total surfactants concentrations are incrementally raised from 2.8% to 3.6%, the full-width at half-maximum (FWHM) of the hydrotalcite diffraction peak diminishes from 0.512° to 0.409°. This reduction in FWHM signifies a more ordered lattice arrangement and an enhanced level of crystallinity within the MgFe-LDHs. However, when total surfactants concentrations of 4.4% is introduced, the FWHM reaches a maximum value of 0.607°. This phenomenon can be attributed to the non—uniform mixing of the micelle solution and the salt solution under conditions of high surfactant concentration [32]. Such non-uniform mixing disrupts the normal crystal growth process, leading to a less ordered structure and a broader FWHM. Based on these observations, it can be concluded that the optimal MgFe-LDH, characterized by the highest surface area and large pores, can be obtained when the wormlike total surfactants concentrations exceed 3.6% and NaCl is used as an inducer. This optimal condition ensures a well-ordered crystal structure with desirable physicochemical properties.

### 2.6. Absorption Experiment

**Figure 12:**
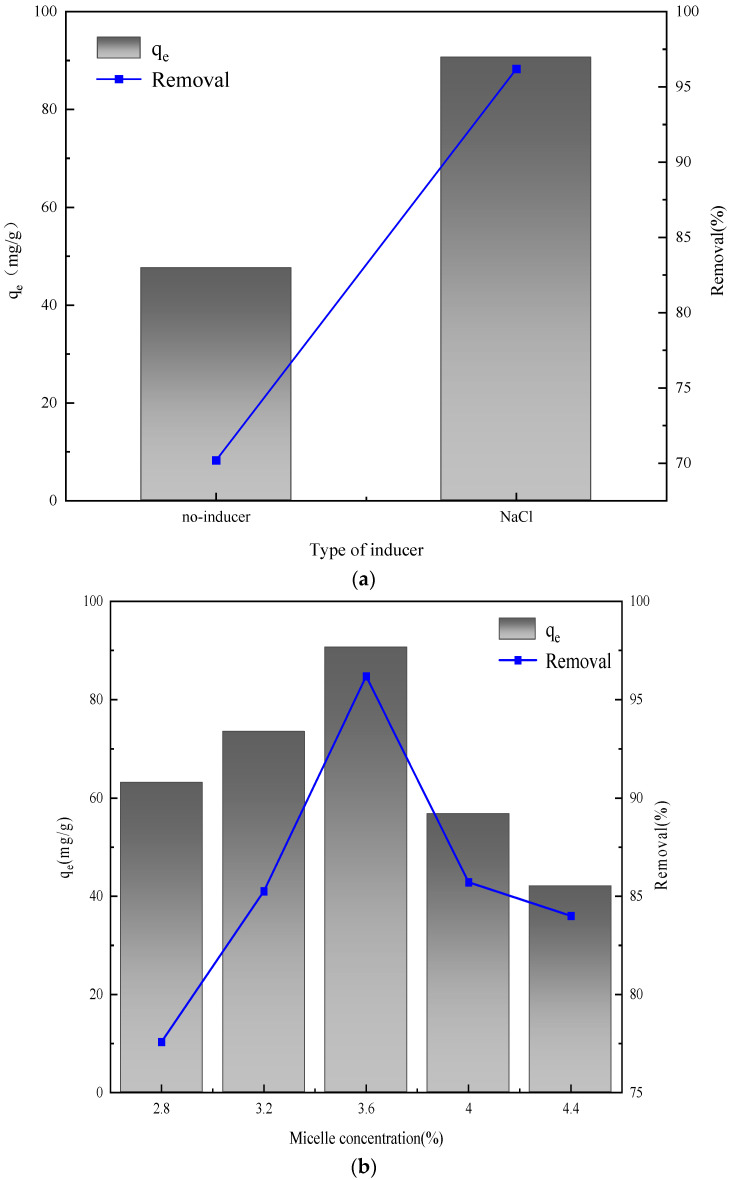
Effect of inducer type (**a**) and total surfactants concentrations (**b**) on the adsorption properties of hydrotalcite.

The adsorption performance of hierarchical layered double hydroxides (LDH) towards sulfonated lignite was investigated under specific experimental conditions: a temperature of 25 °C, a pH of 7, an adsorbent dosage of 0.8 g/L, and a sulfonated lignite concentration of 100 mg/L. As presented in Figure 12, it is evident that MgFe LDH exhibited remarkable adsorption capacity and an efficient removal rate, both reaching 96.19% for sulfonated lignite using NaCl as inducer at 3.6% micelle concentration, which greatly exceed the performance of activated carbon under similar conditions as reported by [33]. This outstanding adsorption performance can be attributed to the superior crystallinity and hierarchical structure of the LDH, which were achieved through the use of wormlike micelles as templates. The hierarchical architecture of the LDH, facilitated by the wormlike micelle templates, provides an enhanced micro-environment that significantly promotes the diffusion of large organic waste molecules, such as those present in sulfonated lignite, during the heterogeneous reaction process. This improved diffusion enables more effective interaction between the LDH and the sulfonated lignite, thereby leading to the high adsorption capacity and removal rate observed.

**Figure 13:**
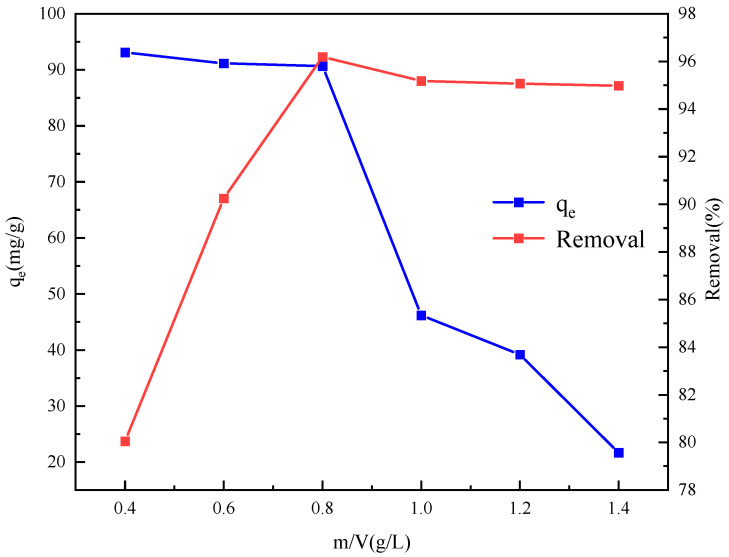
Effect of adsorbent dosage on adsorption performance.

An investigation was carried out to assess the impact of varying adsorbent dosages (0.4, 0.6, 0.8, 1.0, 1.2, and 1.4 g/L) on the adsorption performance for a sulfonated lignite solution with an initial concentration of 100 mg/L. As illustrated in Figure 13, it is evident that the removal efficiency of sulfonated lignite exhibited an upward trend as the quantity of the adsorbent increased. The removal efficiency ultimately attained a relatively stable plateau at 96.0%, which is indicative of the establishment of adsorption equilibrium. However, an interesting phenomenon was observed when the adsorbent dosage reached 1.0 g/L. At this point, the residual concentration of sulfonated lignite in the solution showed a decreasing tendency, dropping from 90.68 mg/L to 46.19 mg/L. This decline can be attributed to the presence of an excessive number of active sites on the adsorbent surface at higher dosages. When there are too many active sites, some of them may remain unsaturated or interact in a non-optimal way, potentially leading to a less efficient utilization of the adsorbent and affecting the overall adsorption process.

**Figure 14:**
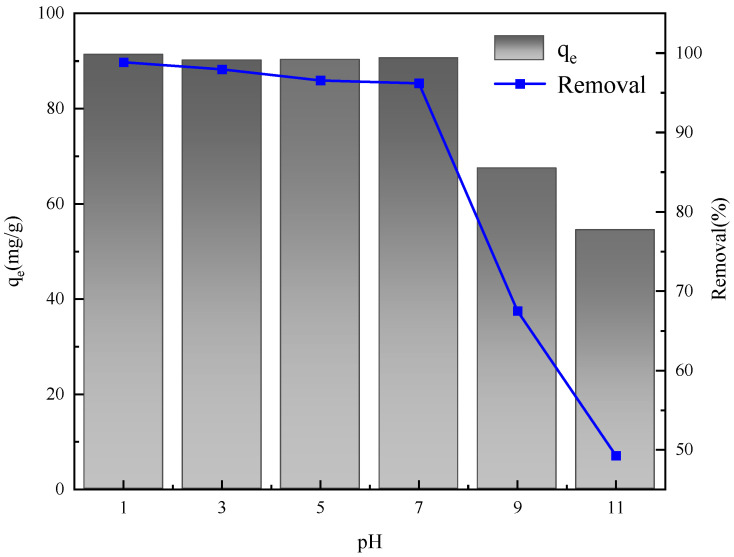
Effect of solution pH on adsorption performance.

During the adsorption process, the pH value plays a pivotal role in determining the extent of protonation of the functional groups present on the adsorbent’s surface, which in turn influences the surface charge of the adsorbent in aqueous solutions with varying pH levels [34]. In this research, the influence of solution pH on the adsorption performance was systematically examined under the following experimental conditions: a temperature of 25 °C, a sulfonated lignite concentration of 100 mg/L, and an adsorbent dosage of 0.8 g/L. The experimental results, as presented in Figure 14, reveal that the adsorption of sulfonated lignite by hydrotalcite is enhanced under acidic conditions. In an acidic environment, a substantial number of H^+^ ions are adsorbed onto the hydrotalcite surface, leading to surface protonation. This protonation process imparts a positive charge to the hydrotalcite surface, thereby facilitating electrostatic attraction between the positively charged surface and the negatively charged sulfonate ions of sulfonated lignite. On the contrary, under alkaline conditions, sulfonated lignite undergoes partial ionization, generating anions such as SO_4_^2−^. The presence of these anions gives rise to competitive adsorption phenomena, where the sulfonated lignite molecules and the sulfonate ions compete for the available adsorption sites on the hydrotalcite surface [35]. This competitive adsorption reduces the overall adsorption efficiency of sulfonated lignite by hydrotalcite under alkaline conditions.

### 2.7. Adsorption Kinetics Analysis

**Figure 15:**
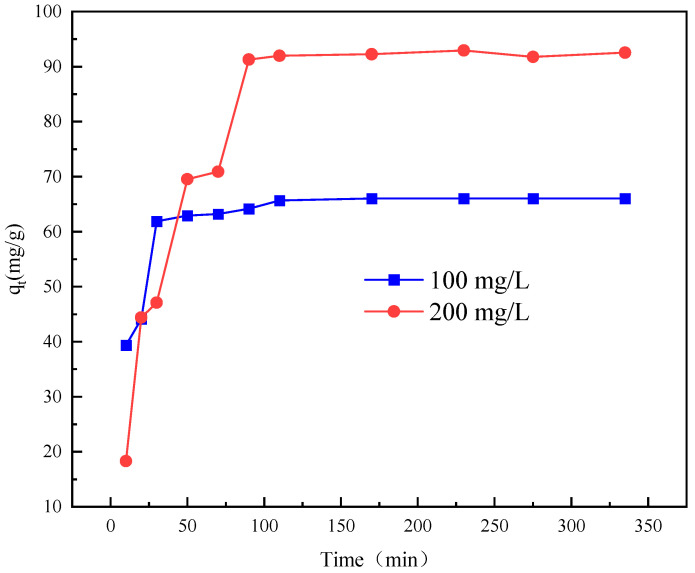
Effect of contact time on adsorption performance.

The adsorption performance of W-MgFe-LDH towards sulfonated lignite was studied under controlled conditions of 25 °C and a pH of 7, with an adsorbent dosage of 0.8 g/L and varying initial concentrations of sulfonated lignite (100 mg/L and 200 mg/L). As depicted in Figure 15, for both initial concentrations, the adsorption process exhibited an upward trend over time. This is primarily attributed to the ample availability of vacant active sites on the adsorbent’s surface at the beginning of the adsorption process. These vacant sites provide numerous opportunities for the sulfonated lignite molecules to attach, leading to an accelerated rate of adsorption. As the adsorption progresses, the number of available active sites gradually decreases until it reaches a point where the adsorption rate levels off and attains a constant value, which is indicative of the establishment of adsorption equilibrium [36].

To gain a deeper insight into the kinetic features of the adsorption process, the experimental data were subjected to fitting with four distinct kinetic models: the pseudo- first-order, pseudo-second-order, liquid film diffusion, and intra-particle diffusion kinetic models. The outcomes of the fitting procedures, along with the corresponding kinetic parameters, are presented in Figure S1 and Table 2. Upon analyzing the results, it becomes evident that the linear fitting curves and the correlation coefficients (R^2^) for both the pseudo-first-order and pseudo-second-order kinetic models demonstrate a high degree of correlation. Specifically, the pseudo-second-order kinetic model exhibits a strong correlation with a value of 0.99. When comparing the linear correlation coefficients of the intra-particle diffusion and liquid film diffusion kinetic models, it is found that the intra-particle diffusion model has a more precise fit. The linear correlation coefficient for the intra-particle diffusion model is approximately 0.85, which is higher than that of the liquid film diffusion model. This finding suggests that the intra-particle diffusion model represents the rate-controlling step in the adsorption process [37].

### 2.8. Analysis of Adsorption Thermodynamics

**Figure 16:**
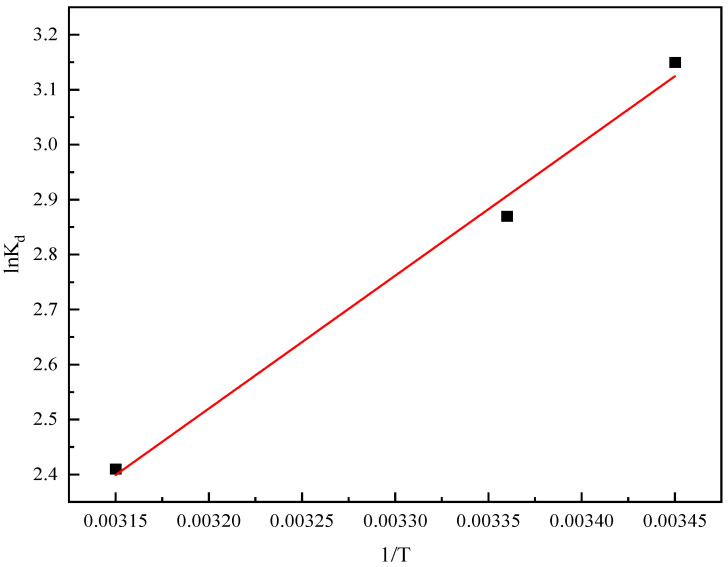
The 1/T fitting curve of lnKd of sulfonated lignite adsorbed by W-MgFe-LDH.

Thermodynamic parameters associated with the adsorption process were ascertained across a range of temperatures. The fitting of isotherms, as illustrated in Figure 16, served as the basis for calculating the corresponding thermodynamic parameters. As presented in Table 3, the standard Gibbs free energy change (ΔG) is found to be negative. This negative value of ΔG is a clear indication that the adsorption process occurs spontaneously. Such a result substantiates the feasibility and inherent spontaneity of the interaction between sulfonated lignite and hierarchical hydrotalcite during the adsorption process [38]. Moreover, the negative values of both the standard enthalpy change (ΔH) and the entropy change (ΔS) provide further insights. The negative ΔH implies that the adsorption process is exothermic in nature, with a reduction in heat release as the process proceeds. Meanwhile, the negative ΔS suggests a decrease in the degree of randomness at the liquid-solid interface during the adsorption event [39].

### 2.9. Adsorption Isotherm Model

At reaction temperatures of 298.15 K and 303.15 K, an investigation into the adsorption isotherm model was conducted utilizing hierarchical porous hydrotalcite W-MgFe-LDH. The adsorbent dosage was set at 0.8 g/L, and the initial concentration of the adsorbate ranged from 150 to 350 mg/L. Three well-established adsorption isotherm models, namely the Langmuir, Freundlich, and Dubin–Redushkevich models, were employed for this study. As evidenced by the results presented in Figure S2 and Table 4, the Langmuir adsorption isotherm model demonstrates a superior fit to the experimental data. This alignment suggests that the adsorption reaction predominantly occurs through the formation of a single-layer coverage on the adsorbent surface. In this scenario, interactions take place between the adsorbate molecules and the vacant adsorption sites on the adsorbent, and the system reaches an equilibrium state that is governed by the rates of adsorption and desorption [40]. Furthermore, based on the Dubin–Redushkevich adsorption isotherm model, the average free energy values at 298 K and 303 K were calculated. These values were found to be less than 8 kJ/mol. Such low free energy values indicate that the adsorption process is primarily controlled by van der Waals forces or molecular electrostatic interactions. This, in turn, reflects a physical adsorption mechanism, where the adsorbate molecules are held onto the adsorbent surface through relatively weak intermolecular forces [41].

### 2.10. Adsorption Competition Experiment

**Figure 17:**
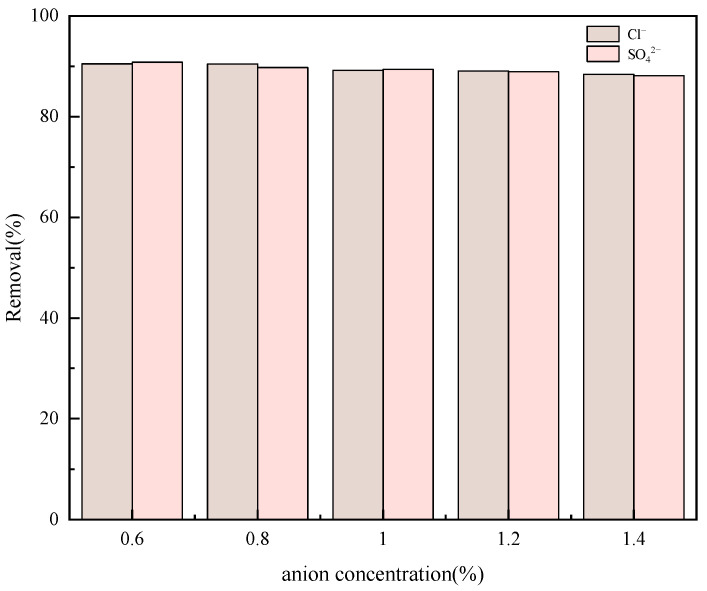
Effect of Cl^−^ and SO_4_^2−^ on adsorption reactions.

Given that the adsorption mechanism underlying this process is predominantly governed by electrostatic attraction, competitive adsorption experiments were incorporated to probe the influence of anions present in the solution on the adsorbent’s capacity to adsorb sulfonated lignite. As this research is primarily centered around an oilfield wastewater system, the study specifically focused on examining the impacts of Cl^−^ and SO_4_^2−^ ions on the adsorption of sulfonated lignite. To conduct this investigation, 0.01 g of sulfonated lignite was dissolved in NaCl and Na_2_SO_4_ solutions with varying mass concentrations (0.6%, 0.8%, 1.0%, 1.2%, and 1.4%). The adsorbent dosage was maintained at 0.8 g/L throughout the experiments. The adsorption of sulfonated lignite by the adsorbent was then assessed at room temperature and a pH of 7. As illustrated in Figure 17, an increase in the anion concentration within the solution was observed to somewhat diminish the adsorbent’s ability to adsorb sulfonated lignite, as these anions interfered with the adsorption reaction. Nevertheless, even at an anion concentration of 1.4%, the removal rate of sulfonated lignite remained relatively high, approximately 90%. This finding underscores the significant potential and desirability of employing hierarchical hydrotalcite as an adsorbent for the removal of sulfonated lignite from water.

### 2.11. Analysis of Recycling

**Figure 18:**
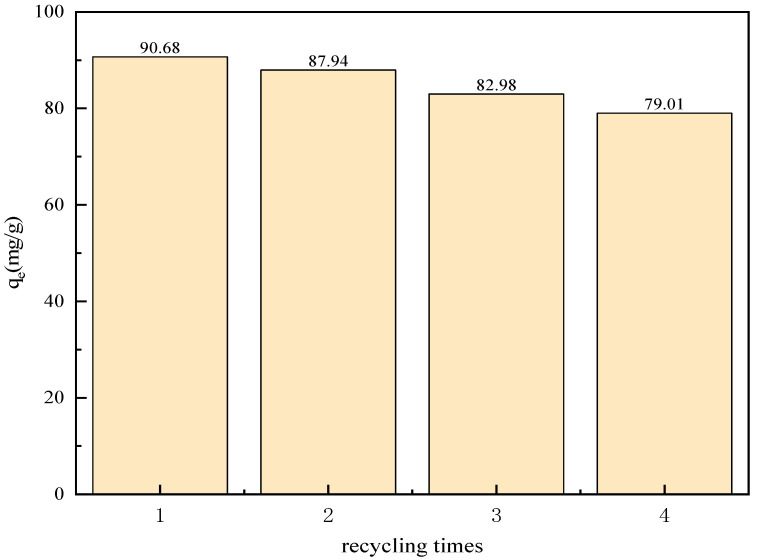
Reusability of hierarchical MgFe hydrotalcite.

**Figure 19:**
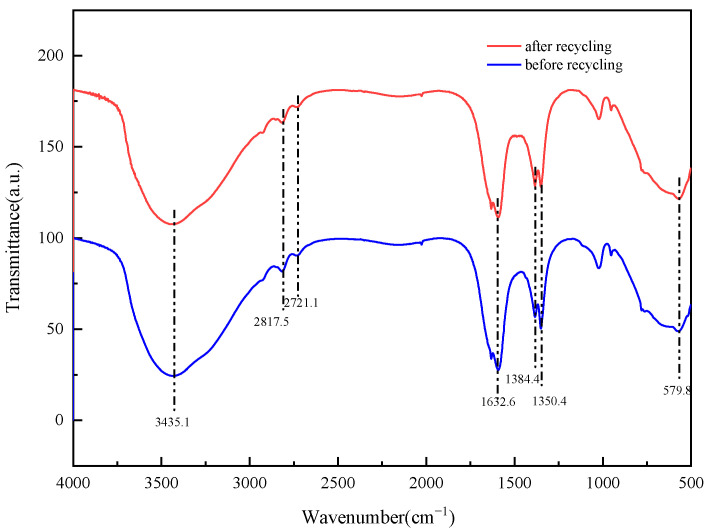
Infrared spectra of before and after regeneration.

**Figure 20:**
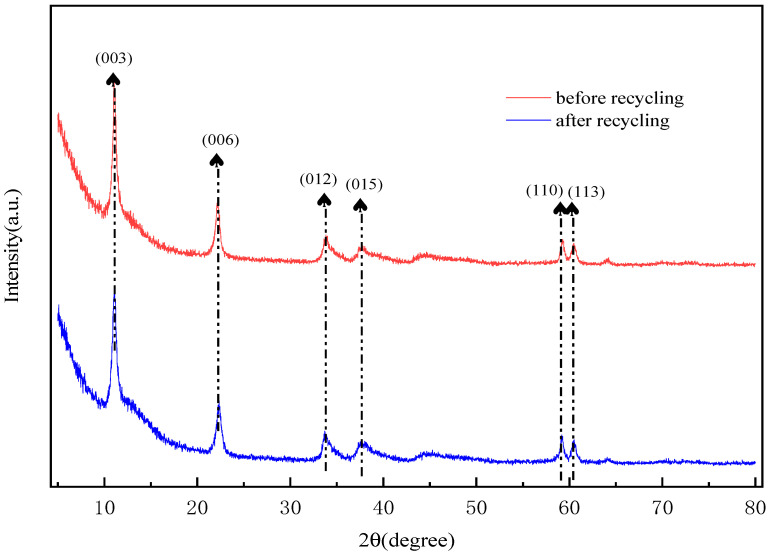
XRD patterns of before and after regeneration.

An investigation was carried out on the recycled adsorbent, which was subjected to three successive regeneration cycles. These cycles were conducted at a temperature of 25 °C, with the solution containing 100 mg/L of sulfonated lignite and an LDH (layered double hydroxide) dosage of 0.8 g/L. At the conclusion of these cycles, it was observed that the adsorption capacity of the adsorbent exhibited a decline, dropping from an initial value of 90.68 mg/g to a final value of 79.01 mg/g, as clearly shown in Figure 18. The results from FT-IR (Fourier-Transform Infrared Spectroscopy) analysis remained consistent across the different cycles, indicating that the active species present on the LDH surface remained unchanged, as shown in Figure 19. However, the XRD (X-Ray Diffraction) patterns revealed a slight decrease in intensity for the reused catalyst, as shown in Figure 20. This suggests that while the active species on the LDH surface were preserved, the pores and the layered structure of the LDH suffered a certain degree of damage. This damage was likely caused by the deposition of sulfonated lignite during the adsorption-regeneration process.

### 2.12. Adsorption Mechanism of Hierarchical MgFe-LDH

**Figure 21:**
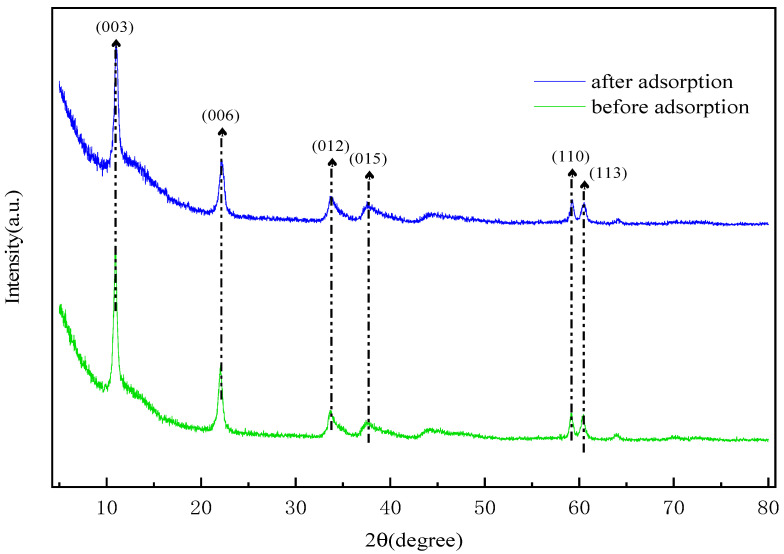
XRD spectra before and after adsorption of adsorbent.

**Figure 22:**
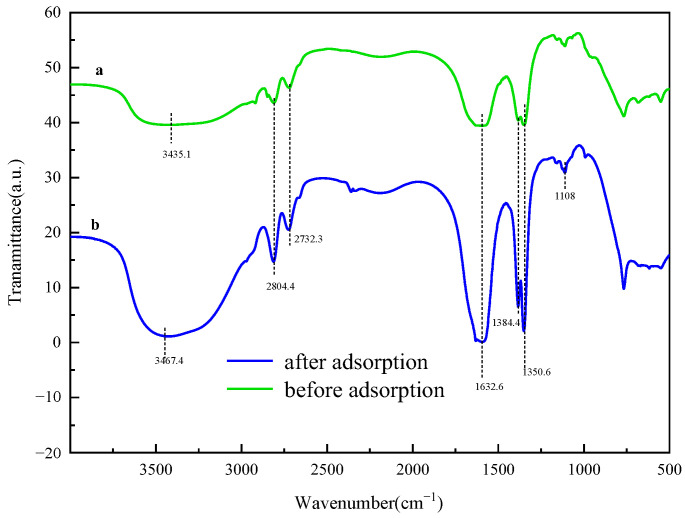
Infrared spectrum before and after adsorption of adsorbent.

**Figure 23:**
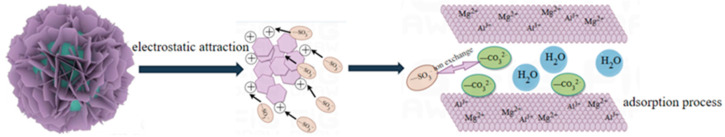
Adsorption mechanism diagram.

Based on the X-Ray Diffraction (XRD) spectra obtained before and after the adsorption process (as shown in Figure 21), it is clearly observable that the material, after adsorbing sulfonated lignite, still exhibits the characteristic diffraction peaks of hydrotalcite. However, a comparison reveals that the peak intensity of the hydrotalcite post-adsorption is slightly lower than that of the pristine hydrotalcite. This reduction in peak intensity implies a decrease in the content of the crystalline phase, which is likely attributable to the adsorption of sulfonated lignite onto the hydrotalcite surface [42]. To gain a more in-depth understanding of the adsorption mechanism of the sulfonated lignite solution on hierarchical MgFe-LDH, Fourier-Transform Infrared Spectroscopy (FTIR) (as shown in Figure 22) and XRD analyses were carried out on the material both before and after adsorption. The results from these analyses indicate that the surface structure of the hierarchical porous hydrotalcite remains largely intact, with only minor changes occurring in the functional groups. Specifically, in the adsorbed sample, asymmetric stretching vibrations of sulfite were detected at 1120.5 cm^−1^, providing conclusive evidence of the successful adsorption of sulfonated lignite onto the W-MgFe-LDH surface. Moreover, the O-H stretching vibration band shifted from 3435.1 cm^−1^ to 3467.4 cm^−1^. This shift suggests the formation of ligand bonds between functional groups (such as hydroxyl and carboxyl groups) within the sulfonated lignite molecule and the hydroxyl groups present on the W-MgFe-LDH surface [43]. In conclusion, as depicted in Figure 23, the adsorption mechanism of MgFe-LDH towards sulfonated lignite involves electrostatic attraction. Negatively charged sulfonate ions, generated through the ionization of sulfonated lignite, are attracted to the positively charged ions on the hydrotalcite surface, facilitating electrostatic adsorption. Furthermore, due to the interlayer anion-exchange capacity of hydrotalcite, the adsorbed sulfonate ions can undergo an exchange reaction with interlayer carbonate or hydroxide anions. This anion-exchange process serves to drive the adsorption process forward [44].

## 3. Conclusions

In this study, a hierarchical structure was achieved in MgFe-LDH through co-precipitation over worm like micelle soft template by using sodium chloride (NaCl) as an additive to promote the self-assembly of zwitterionic oleyl aminopropyl betaine (OAPB) and anionic sodium dodecyl sulfate (SLS). The prepared hierarchical hydrotalcite exhibited significant adsorption potential for aqueous solutions containing sulfonated lignite. The material has an adsorption capacity of 90.68 mg/g for sulfonated lignite solution, which is superior to that of traditional LDH. The hybrid micellar template played a crucial role in determining the morphology and pore structure of MgFe-LDH nanoparticles. Furthermore, the adsorption of sulfonated lignite followed a pseudo-second-order kinetic equation with intra-particle diffusion identified as the rate-limiting step. The adsorption isotherms were consistent with the Langmuir model, indicating monolayer adsorption of sulfonated lignite. Thermodynamic analysis revealed an exothermic and spontaneous adsorption process driven mainly by electrostatic attraction between graded MgFe-LDH and sulfonated lignite. Based on it, a straightforward and effective method for preparing high-performance materials with excellent capability of efficiently removing macromolecular organic pollutants from oilfield wastewater was established.

## 4. Materials and Methods

### 4.1. Reagents

All reagents employed in this study, namely sodium dodecyl sulfate (SLS, purity: 99.0%), oleyl amidopropyl betaine (OAPB, purity: 99.0%), sodium hydroxide (NaOH, purity: 99.0%), anhydrous sodium carbonate (Na_2_CO_3_, purity: 99.0%), sodium chloride (NaCl, purity: 99%), and absolute ethanol, were of analytical grade and utilized without additional purification steps. These chemical substances were procured from Tianjin Damao Chemical Reagent Factory, located in Tianjin, China. The sulfonated lignite utilized in the research was sourced from Tarim, Xinjiang, China. Deionized water was consistently employed for the preparation of solutions and the rinsing of precipitates across all experimental procedures.

### 4.2. Preparation of Absorbents

**Figure 24:**
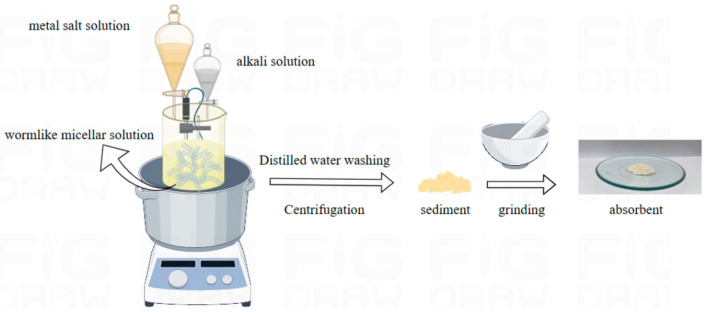
Experimental process diagram.

The wormlike micelles solution was prepared by dissolving OAPB and SLS at a 3:1 molar ratio in 0.1 mol/L NaCl aqueous solution. And then metal salt solution and the alkali solution were added dropwise to wormlike micelles at 45 °C under a pH of 7 for 15 h under agitation. After centrifugation the precipitate was dried and ground to obtain hierarchical hydrotalcite (W-MgFe-LDH) as illustrated in Figure 24.

### 4.3. Adsorption Experiments

To assess the removal efficacy of W-MgFe-LDH (hierarchical magnesium-iron layered double hydroxide) towards sulfonated lignite (SL) in an aqueous environment, a series of batch adsorption experiments were systematically carried out. A predetermined quantity of W-MgFe-LDH was introduced into 100 mL of an aqueous solution with a known concentration of SL (100 mg/L). These experiments were conducted at a controlled temperature of 25 °C, with continuous stirring maintained at a constant speed of 200 revolutions per minute (rpm). Upon reaching the adsorption equilibrium state, the adsorbent material was carefully separated from the solution. The residual concentration of SL in the solution was subsequently quantified utilizing a UV-Vis spectrophotometer (Model: UV-4802S, manufactured by Unico, Reno, NV, USA), with measurements taken at the peak absorbance wavelength of 320 nm. The adsorption capacity at any given time t, denoted as *q_t_* (in units of mg/g), the equilibrium adsorption capacity, *q_e_* (also in mg/g), along with the percentage removal efficiency of SL were computed based on the following mathematical formulations:

(1) $$q_{t}=\frac{\left(c_{0}-c_{t}\right)\times V}{m}$$

(2) $$q_{e}=\frac{\left(c_{0}-c_{e}\right)\times V}{m}$$

(3) $$Removal=\frac{c_{0}-c_{e}}{c_{0}}\times 100\%$$

### 4.4. Recycling Experiments

The used W-MgFe-LDH samples were subjected to repeated adsorption cycles after desorption tests at a constant temperature of 25 °C under the same reaction conditions of 100 mg/L SL solution 1.0 g/L of adsorbent.

### 4.5. MgFe-LDH Characterization

XRD patterns were collected at room temperature using an X-ray diffractometer with Cu Kα radiation in the range of 5° ≤ 2θ ≤ 80°. The Micromeritics ASAP 2020 instrument (Norcross, GA, USA) was used to measure nitrogen adsorption and desorption isotherms. BET method and Barrett–Joyner–Halenda (BJH) model were used to calculate the surface area, the pore size distribution and pore structure, respectively. Weight loss TG curves were recorded under air by a Thermo-gravimetric analyzer (TG-SDTA851) with a heating rate of 10 °C/min from room temperature to 1000 °C. Using a scanning electron microscope with a 20.0 kV accelerating voltage (SEM, JSM-6390A, JEOL, Tokyo, Japan) the morphology of the prepared material was elucidated. FTIR spectra were collected using a Nicolet 5700 Fourier transform infrared (Thermo Electron Co., West Palm Beach, FL, USA) and 1 mg of the sample was homogenized with 200 mg of KBr to form powder pellets.

### 4.6. Rheological Characterization

Steady-state and dynamic rheological properties of micellar systems were analyzed using rheological testing methods. The apparent viscosity variation of 0.1 wt% PAM-AMPS/Fe_3_O_4_ nanocomposite fluids was measured using a coaxial cylindrical ro-tor system (CC39 open-top) across a shear rate range of 0.1–1000 s^−1^, revealing their shear-thinning behavior. Concurrently, dynamic viscoelasticity testing was conducted using a cone-plate rotor system (CP50): First, a strain scan from 0.1% to 100% at a fixed frequency of 1 Hz was performed to determine the boundary of the linear viscoelastic region. Subsequently, specific strain values within this region were selected to obtain the frequency-dependent characteristics of the storage modulus (G′), loss modulus (G″), and complex viscosity (η) through wide-frequency-domain dynamic oscillation experiments (0.1–100 rad/s). This approach systematically characterized the viscoelastic relaxation dynamics and microstructural stability of the micellar network.

## Figures and Tables

**Table 1:** Brunauer–Emmett–Teller (BET) surface area, pore volume, and pore diameter of the different MgFe-LDH nanoparticles.

Sample	Specific Area (m^2^/g)	Pore Volume (cm^3^/g)	Pore Diameter (nm)
ordinary hydrotalcite	110.23	0.63	28.2
multi-porous hydrotalcite	199.82	0.78	12.7

**Table 2:** Parameters of four kinetic models.

Kinetic Model	Parameter	Concentration (mg/L)	
		100	200
pseudo-first-order	q_e_ (mg/g) model	29.37	156.02
	K_1_ (h^−1^)	0.037	0.044
	R^2^	0.85	0.87
pseudo-second-order	q_e_ (mg/g) model	66.67	102.66
	q_e_ (mg/g) experiment	66.02	92.95
	K_2_ (g/m gh)	0.0029	0.00036
	R^2^	0.99	0.99
Intraparticle diffusion model	K_i1_ (mg/gh^1/2^)	9.42	10.04
	R_1_^2^	0.70	0.92
	K_i2_ (mg/gh^1/2^)	0.70	1.23
	R_2_^2^	0.89	0.34
Liquid film diffusion model	K_fd_ (h^−1^)	0.037	0.044
	R^2^	0.85	0.87

**Table 3:** Thermodynamic parameters of W-MgFe-LDH adsorbing sulfonated lignite.

T (K)	∆S [J/(mol·k)]	∆H [kJ/mol]	∆G (kJ/mol)	R^2^
298.15	−43.53	−20.09	−7.11	0.992
303.15			−6.89	
313.15			−6.29	

**Table 4:** Isothermal model parameters of W-MgFe-LDH adsorbing sulfonated lignite.

Isothermal Adsorption Model	Parameter	Temperature	
		298.15 K	303.15 K
Langmuir	q_m_ (mg/g)	73.48	70.32
	b (mg/L)	3333.33	2777.78
	R^2^	0.99	0.99
Freundlich	K_f_ (L/g)	33,884.42	40,738.03
	n	−18.18	−13.33
	R^2^	0.96	0.48
D-R	q_m_ (mg/g)	403.97	396.47
	β (mol^2^/kJ^2^)	0.42	0.21
	R^2^	0.89	0.29
	E (kJ/mol)	1.09	1.54

## Data Availability

The original contributions presented in this study are included in the article/Supplementary Material. Further inquiries can be directed to the corresponding authors.

## Supplementary Materials

The following supporting information can be downloaded at: https://www.mdpi.com/article/10.3390/gels11090714/s1, Figure S1: Four kinetic models (a. pseudo-first-order model; b. pseudo-second-order model; c. intraparticle diffusion kinetic model; d. liquid film diffusion model); Figure S2: Three adsorption isotherm models (a. Langmuir model; b. Freundlich model; c. D-R model).

## References

[1] Shen H., Zhang W.Y. (2018). Synthesis of Lignite Graft Polycondensate as Drilling Fluid Additive and its Influence on the Properties of Water-Bentonite Suspensions. Chem. Technol. Fuels Oils.

[2] Zhang S.X., Wang Z.H., Lü N.C., He H.J., Wang Z.M., Yang Z.G. (2015). Physicochemical and microbial treatment technology for polysulfonate drilling fluid wastewater. Chin. J. Environ. Eng..

[3] Guo D.W., Wu J.B., Feng D.D., Zhang Y., Zhu X., Luo Z., Kang Y., Zhao Y., Sun S. (2023). Mechanism of efficient magnetic biochar for typical aqueous organic contaminant combined-adsorption removal. Fuel Process. Technol..

[4] Dehmani Y., Ba M.B., Oukhrib R., Dehbi A., Lamhasni T., Brahmi Y., El-Kordy A., Franco D.S., Georgin J., Lima E.C. (2024). Adsorption of various inorganic and organic pollutants by natural and synthetic zeolites: A critical review. Arab. J. Chem..

[5] Chen C.L., Ma J.Y., Wang Y., Yi Z., Wang S., Gao H., Wu X., Liu G., Yang H. (2023). CTAB-assisted synthesis of Bi_2_MoO_6_ hierarchical microsphere and its application as a novel efficient and recyclable adsorbent in removing organic pollutants. Colloids Surf. A Physicochem. Eng. Asp..

[6] Ukalska-Jaruga A., Bejger R., Smreczak B., Podlasiński M. (2023). Sorption of Organic Contaminants by Stable Organic Matter Fraction in Soil. Molecules.

[7] Wu H.F., Chao Y.H., Jin Y., Tao D., Li X., Luo J., Xia G., Zhu L., Zhu W. (2022). Sustainable preparation of graphene-analogue boron nitride by ball-milling for adsorption of organic pollutants. Chin. J. Chem. Eng..

[8] Fernandez D.C., Morales D.S., Jiménez J.R., Fernández-Rodriguez J.M. (2022). CO_2_ adsorption by organohydrotalcites at low temperatures and high pressure. Chem. Eng. J..

[9] Shu P., Zhang Y.N., Deng J., Duan Z., Zhai F. (2023). Characteristics and mechanism of modified hydrotalcite for coal spontaneous combustion preventing. Energy.

[10] Ogata F., Nagai N., Kishida M., Nakamura T., Kawasaki N. (2019). Interaction between phosphate ions and Fe-Mg type hydrotalcite for purification of wastewater. J. Environ. Chem. Eng..

[11] Avendaño R., Fals J., Bocanegra S., Dieuzeide M.L., Amadeo N. (2023). Sorption enhanced steam reforming of ethanol for hydrogen production, over Mg/Al hydrotalcites modified with K. Int. J. Hydrogen Energy.

[12] López-Santiago R.F., Delgado J., Castillo R. (2022). Micellar entanglement and its relation to the elastic behavior of wormlike micelle fluids. J. Colloid Interface Sci..

[13] Stancheva T.N., Georgiev M.T., Radulova G.M., Danov K.D., Marinova K.G. (2022). Rheology of saturated micellar networks: Wormlike micellar solutions vs. bicontinuous micellar phases. Colloids Surf. A Physicochem. Eng. Asp..

[14] Mushi S.J., Kang W., Yang H., Li Z., Ibrashev K., Issakhov M., Mabeyo P.E. (2021). Effect of aromatic acid on the rheological behaviors and microstructural mechanism of wormlike micelles in betaine surfactant. J. Mol. Liq..

[15] Wu A.L., Gao Y.N., Zheng L.Q. (2019). Zwitterionic amphiphiles: Their aggregation behavior and applications. Green Chem..

[16] Lu S., Dong J.F., Li X.F. (2023). Gradual transformation of anionic/zwitterionic wormlike micelles from viscous to elastic domains: Unravelling the effect of anionic surfactant chain length. J. Colloid Interface Sci..

[17] Molchanov V.S., Kuklin A.I., Orekhov A.S., Arkharova N., Philippova O. (2021). Temporally persistent networks of long-lived mixed wormlike micelles of zwitterionic and anionic surfactants. J. Mol. Liq..

[18] Qiao Y., Lin Y.Y., Wang Y.J., Li Z., Huang J. (2011). Metal-Driven Viscoelastic Wormlike Micelle in Anionic/Zwitterionic Surfactant Systems and Template-Directed Synthesis of Dendritic Silver Nanostructures. Langmuir.

[19] Lu S., Mei Q.L., Chen J.Y., Wang Z., Li W., Feng C., Li X., Dong J. (2022). Cryo-TEM and rheological study on shear-thickening wormlike micelles of zwitterionic/anionic (AHSB/SDS) surfactants. J. Colloid Interface Sci..

[20] Wang X.Q., Wang R.T., Zheng Y., Sun L., Yu L., Jiao J., Wang R. (2013). Interaction between Zwitterionic Surface Activity Ionic Liquid and Anionic Surfactant: Na^+^-Driven Wormlike Micelles. J. Phys. Chem. B.

[21] Li Y., Gao Q.Y. (2023). Novel self-assembly nano OSA starch micelles controlled by protonation in aqueous media. Carbohydr. Polym..

[22] Yang J.W., Wu T.J., Liu Q.N., Huang H., Chen S., Chen G. (2024). Research of a fracturing-oil displacement integrated working fluid based on betaine surfactant. Colloids Surf. A Physicochem. Eng. Asp..

[23] Rothstein J.P., Mohammadigoushki H. (2020). Complex flows of viscoelastic wormlike micelle solutions. J. Non-Newton. Fluid Mech..

[24] Tamate R., Hashimoto K., Li X., Shibayama M., Watanabe M. (2019). Effect of ionic liquid structure on viscoelastic behavior of hydrogen-bonded micellar ion gels. Polymer.

[25] Mallakpour S., Hatami M., Hussain C.M. (2020). Recent innovations in functionalized layered double hydroxides: Fabrication, characterization, and industrial applications. Adv. Colloid Interface Sci..

[26] Balbino T.A.C., Bellato C.R., da Silva A.D., Neto J.d.O.M., Ferreira S.O. (2020). Preparation and evaluation of iron oxide/hydrotalcite intercalated with dodecylsulfate/β-cyclodextrin magnetic organocomposite for phenolic compounds removal. Appl. Clay Sci..

[27] Gao H.M., Yao A., Shi Y.H., Noor N., Zeb A., Li M., Li H. (2020). Preparation and properties of hierarchical Al–Mg layered double hydroxides as UV resistant hydrotalcite. Mater. Chem. Phys..

[28] Alaei R., Javanshir S., Behnamfard A. (2020). Treatment of gold ore cyanidation wastewater by adsorption onto a Hydrotalcite-type anionic clay as a novel adsorbent. J. Environ. Health Sci. Eng..

[29] DVelázquez-Herrera F., Fetter G., Rosato V., Pereyra A.M., Basaldella E.I. (2018). Effect of structure, morphology and chemical composition of Zn-Al, Mg/Zn-Al and Cu/Zn-Al hydrotalcites on their antifungal activity against *A. niger*. J. Environ. Chem. Eng..

[30] Comelli N.A., Ruiz M.L., Leguizamón Aparicio M.S., Merino N.A., Cecilia J.A., Rodríguez-Castellón E., Lick I.D., Ponzi M.I. (2018). Influence of the synthetic conditions on the composition, morphology of CuMgAl hydrotalcites and their use as catalytic precursor in diesel soot combustion reactions. Appl. Clay Sci..

[31] Vu T., Weaver M.R., Kasting G.B., Koenig P. (2021). Effect of pH on the Structure and Dynamics of Wormlike Micelles in an Amino Acid-Derived Surfactant Composition. Langmuir.

[32] Zhou L., Slan M., Bai B.B., Du W., Qu C., Zhang J., Tang Y. (2021). Enhanced Removal of Sulfonated Lignite from Oil Wastewater with Multidimensional MgAl-LDH Nanoparticles. Nanomaterials.

[33] Wang W.H., Hu B.B., Wang C., Liang Z., Cui F., Zhao Z., Yang C. (2020). Cr(VI) removal by micron-scale iron-carbon composite induced by ball milling: The role of activated carbon. Chem. Eng. J..

[34] Motta F.L., Melo B.A.G., Santana M.H.A. (2016). Deprotonation and protonation of humic acids as a strategy for the technological development of pH-responsive nanoparticles with fungicidal potential. New Biotechnol..

[35] Bashiri H., Hassani Javanmardi A., Soltani Z. (2024). A theoretical description for competitive adsorption at the Solid/Solution interface. Comput. Theor. Chem..

[36] Wu Z.S., Sun Z.H., Liu P.Y., Li Q., Yang R., Yang X. (2020). Competitive adsorption of naphthalene and phenanthrene on walnut shell based activated carbon and the verification via theoretical calculation. RSC Adv..

[37] Ren N., Wang C.H., Zhao Z., Liang Y., Wei W., Qin G. (2021). Recovery of ferulic acid from corn bran by adsorption on mesoporous carbon. J. Food Process Eng..

[38] Fang Y., Yang K., Zhang Y.P., Peng C., Robledo-Cabrera A., López-Valdivieso A. (2021). Highly surface activated carbon to remove Cr(VI) from aqueous solution with adsorbent recycling. Environ. Res..

[39] Legrand U., Castillo Sánchez J.R., Boudreault R., Meunier J.-L., Lauriault P.-L.G., Tavares J.R. (2022). Fundamental thermodynamic properties of sorbents for atmospheric water capture. Chem. Eng. J..

[40] Niknezhad M., Lakouraj M.M. (2021). Development of pH-sensitive hydrogel nanocomposite based on acrylic acid/graphene oxide/acryloyl tetra ammonium thiacalix[4]arene for separation of cationic dyes. J. Polym. Res..

[41] Koyuncu H., Kul A.R. (2019). Removal of aniline from aqueous solution by activated kaolinite: Kinetic, equilibrium and thermodynamic studies. Colloids Surf. A Physicochem. Eng. Asp..

[42] Chadha N., Sharma R., Saini P. (2021). A new insight into the structural modulation of graphene oxide upon chemical reduction probed by Raman spectroscopy and X-ray diffraction. Carbon Lett..

[43] Burciaga-Díaz O., Escalante-García J.I. (2022). Structural transition to well-ordered phases of NaOH-activated slag-metakaolin cements aged by 6 years. Cem. Concr. Res..

[44] Liu H.Z., Huang P.J., Liang Z.J., Zhao Z., Cui F. (2022). Selective adsorption of anions on hydrotalcite-like compounds derived from drinking water treatment residuals. Chemosphere.

