# Supplementary material for: Synthesis of Porous Materials on Hybrid Wormlike Micelles of Zwitterionic and Anionic Surfactants for Efficient Oilfield Wastewater Treatment
Source: Gels. 2025 Sep 5;11(9):714. doi: 10.3390/gels11090714 (PMC12469700; doi:10.3390/gels11090714)

Synthesis of porous materials on zwitterionic wormlike micelles templates and its application for efficient treatment of oilfield wastewater

Fei Liu[1*], Zhenzhen Li[2], Xiaolong Wan[3], Chenrui Jiang[3], Chenye Yang [2],Ying Tang[2*]

1 College of Petroleum Engineering, Shandong Institute of Petroleum and Chemical Technology, Dongying, Shandong 257061, P. R. China
2. Shaanxi Province Key Laboratory of Environmental Pollution Control and Reservoir Protection Technology of Oilfields, Xi'an Shiyou University, Xi'an,710065, China
3. Oil Production Plant 11 of Chqingqing Oilfield Company, Xi'an China, 710018
Correspondence: 2018009@sdipct.edu.cn; tangying78@xsyu.edu.cn

Supplementary Materials:

Figure S1: Four kinetic models (a. pseudo-first-order model; b. pseudo-second-order model; c. in-traparticle diffusion kinetic model; d. liquid film diffusion model).

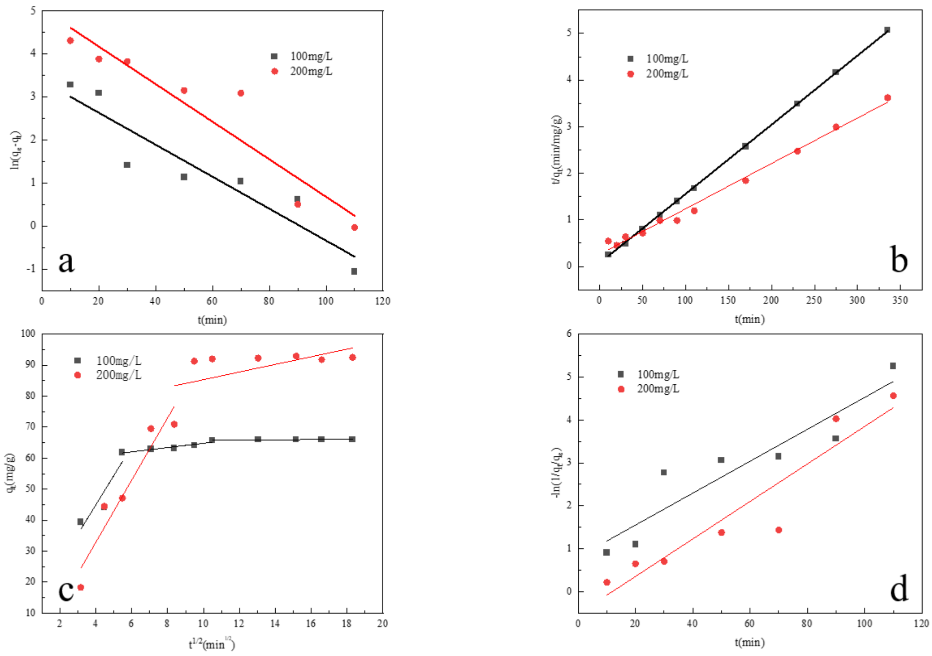

Figure S2: Three adsorption isotherm models (a. Langmuir model; b. Freundlich model; c. D-R model).

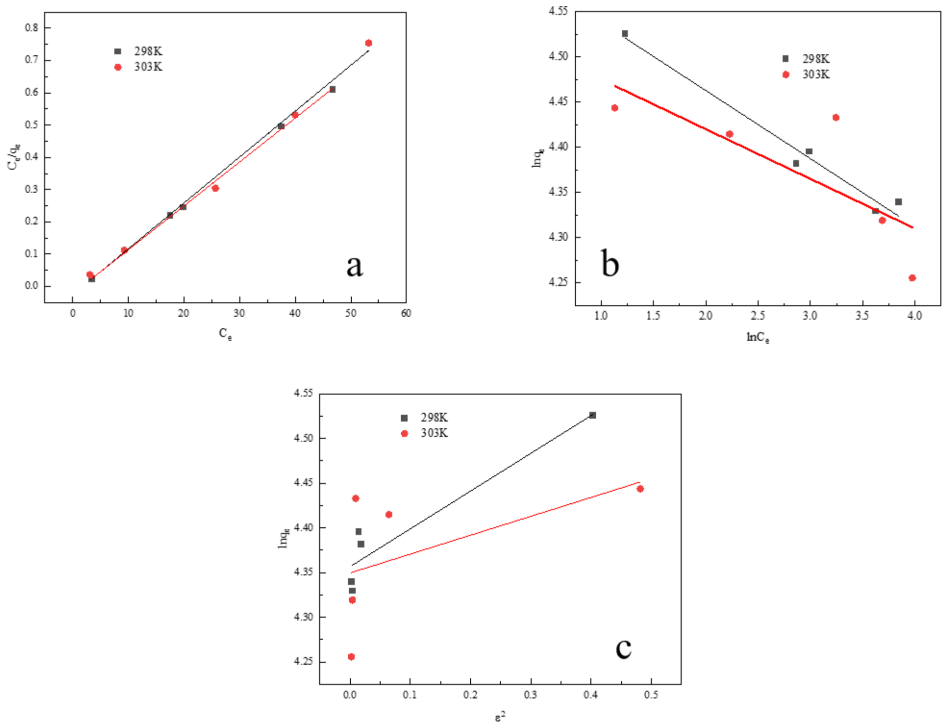

Supplement: Supplementary file 1 [file gels-11-00714-s001.zip › gels-3818395-supplementary.pdf]
